# The neuronal basis of human creativity

**DOI:** 10.3389/fnhum.2024.1367922

**Published:** 2024-02-27

**Authors:** Rafael Malach

**Affiliations:** Department of Brain Sciences, Weizmann Institute of Science, Rehovot, Israel

**Keywords:** cortex, human, spontaneous, resting, creativity

## Abstract

Human creativity is a powerful cognitive ability underlying all uniquely human cultural and scientific advancement. However, the neuronal basis of this creative ability is unknown. Here, I propose that slow, spontaneous fluctuations in neuronal activity, also known as “resting state” fluctuations, constitute a universal mechanism underlying all facets of human creativity. Support for this hypothesis is derived from experiments that directly link spontaneous fluctuations and verbal creativity. Recent experimental and modeling advances in our understanding of the spontaneous fluctuations offer an explanation for the diversity and innovative nature of creativity, which is derived from a unique integration of random, neuronal noise on the one hand with individually specified, deterministic information acquired through learning, expertise training, and hereditary traits. This integration between stochasticity and order leads to a process that offers, on the one hand, original, unexpected outcomes but, on the other hand, endows these outcomes with knowledge-based meaning and significance.

## Introduction

Among the diverse aspects that make humans unique, the most outstanding perhaps is the unparalleled advances humanity has made in science and culture, from inventing space telescopes to creating Shakespeare’s plays, no other species can even remotely match such accomplishments. These spectacular achievements are, of course, a combined result of many human attributes such as language, model building, and recursive thinking. However, a critical element that can be pointed out as a pivotal ingredient underlying all these achievements is human creativity. Without the novel and significant insights of the creative individual, human activity will move in a loop, endlessly repeating prior patterns of behavior. Thus, undoubtedly, human creativity is a unique and decisive engine of progress across human cultural history. Surprisingly, despite its fundamental significance—our understanding of the human brain mechanisms that underlie creative behaviors is so far poor and limited.

This review first proposes a straightforward strategy for searching for such neuronal mechanisms underlying creative behaviors. The idea is to look for those neuronal processes that manifest common and well-accepted aspects of the creative process. Using this “template matching” strategy, we can then proceed to verify the link between the putative neuronal mechanism and creative behaviors using targeted experiments. The following sections illustrate how this approach was taken in a laboratory setting, leading to the identification of a putative universal neuronal mechanism underlying all creative behaviors.

### Identifying key characteristics of human creativity

It is important, prior to any consideration of aspects of creativity, to define what this rather enigmatic process is. Not surprisingly, one may consider numerous different definitions, and these definitions impact how we treat the topic. This review adopts a simple definition that is operationally tractable and intuitive while noting that there may be a number of other possible definitions. Creativity will be defined here as the production of something that is both new and significant. The novelty aspect of creativity is obvious; we will not consider merely copying existing material to be a creative act. However, not everything that is new will necessarily be considered creative; if I scribble a meaningless line on a napkin, it is certainly novel but will not be considered a creative act because it is not significant. For something to be creative, the novel creation should have meaning and have an impact that goes far beyond the created item itself. Therefore, production that is novel and significant will be considered a creative act throughout the article.

Once we define the term, a number of characteristics of creativity become readily apparent. Below, the central characteristics are described.

### Diversity

While we tend to think of human creative insights as rare and unique acts of genius, considering the range of behaviors where we encounter a creative act, one is struck by how diverse and multidimensional these can be. Creative acts appear in the motor domain, e.g., in choreography, in the language domain in poetry and literature, and, of course, in technological and scientific breakthroughs and innovations. In fact, it is hard to consider any domain of human behavior in which creativity cannot manifest itself. Thus, following the overall strategy outlined above, a universal neuronal mechanism that underlies creativity must express itself in all domains of human behaviors, which neuronally translates to being present in all networks of the cerebral cortex, where these diverse domains are likely represented.

### Spontaneity

Following the operational definition of creativity—a creative act cannot be fully controlled by externally delivered stimulations, instructions, or commands, which, by definition, contradicts the novelty aspect. The creative act must include a dominant element of self-generated spontaneity. Thus, when searching for a universal neuronal mechanism underlying creativity, we need to search for a process that is intrinsic and self-generated by the cortex and is not externally driven and fully controlled by outside factors.

### Sub-consciousness

This is likely the less intuitive aspect of the creative process; it is commonly assumed that arriving at a creative insight is a deliberate, intentional process under the full attention and awareness of the creative individual. However, careful introspection reveals that, in general, the process contains a crucial incubation stage that goes on below the awareness threshold. This can be easily demonstrated if we consider the following, quite common, scenario. Imagine you are faced with a difficult problem in your field of expertise. You are bothered by this problem, you are eager to solve it, yet no answer comes to mind. After struggling with this problem for a while, you simply give up. However, a few hours, sometimes days, after these futile attempts, often in a completely irrelevant context, such as while jogging, taking a bath, or even while dreaming or waking up from sleep, suddenly the answer, almost always complete and correct, is fully present in your mind. This surprising and striking “out of the blue” experience of insight can only be explained by a process of search and incubation that goes on subconsciously until the solution comes up. Therefore, a surprising aspect of the neuronal mechanism we should search for when looking for a creativity generator should be that this process is active in the absence of awareness.

### Spontaneous (resting state) fluctuations as a putative generator of creative insights

Exploring possible candidate mechanisms that may fulfill the three aspects outlined above highlights a surprisingly ubiquitous and well-studied neuronal mechanism: ultra-slow fluctuations in cortical activity also termed spontaneous or resting state fluctuations. In the rest of the article, the term “spontaneous fluctuations” will be used to refer to this cortical phenomenon. Spontaneous fluctuations are ultra-slow modulations in neuronal activity mainly studied in the human cerebral cortex, although pioneering work on these spontaneous fluctuations started in animal models (Arieli et al., 1996). The spontaneous fluctuations are widespread across the cortex, obey a power-law spectrum with robust ultra-slow frequencies of 1–5 s periods, and have been described in different modalities such as fMRI (Nir et al., 2006; Fox and Raichle, 2007) and intra-cranial EEG (Nir et al., 2008). They are organized in informative patterns of correlations termed resting-state networks (Smith et al., 2009) that have been studied extensively in recent years. However, despite being a target of a massive research effort, no consensus currently exists as to the role these activity fluctuations may play in cognitive or behavioral function.

Examining the properties of the spontaneous fluctuations shows a remarkable correspondence to the three “signature” characteristics proposed above for a universal creativity-inducing neuronal mechanism. First, these fluctuations are found across the entire extent of the cerebral cortex, in line with the diversity criterion. Second, they are, as their name implies, generated spontaneously and are not dependent on external stimulation or task. Finally, indirect experiments reveal that individuals fail to notice the occurance of the spontaneous fluctuations in their own brains. Thus, in real time neurofeedback experiments, participants, undergoing fMRI scanning, were given an auditory feedback whenever a high amplitude fluctuation appeared in their category-selective visual areas. Depite the clear and consistent association between the spontaneous activation in these visual areas and the auditory signals given to the participants, over several sessions, participants completely failed to note that such an audio-visual link existed. Furthermore, the two visual areas from which the auditory signals were triggered were, in one set of experiments, an area in which typical awareness-related activations lead to an experience of seeing faces, and in another set of experiments, an area in which typical activation leads to seeing places.

However, participants were at chance when specifically asked to guess which of these two cateogry-selective visual areas triggered the auditory signals they heard (Ramot et al., 2016). In another set of studies, the spontaneous fluctuations were linked to slow eye movements that occurred when participants closed their eyes yet were unaware that any such ocular movements and fluctuations were actually happening (Ramot et al., 2011). Based on such indirect evidence, one can conclude that the spontaneous fluctuations go on below the individual’s awareness threshold [see also Moutard et al. (2015)].

### The functional role of spontaneous fluctuations in the creative process

**Figure 1:**
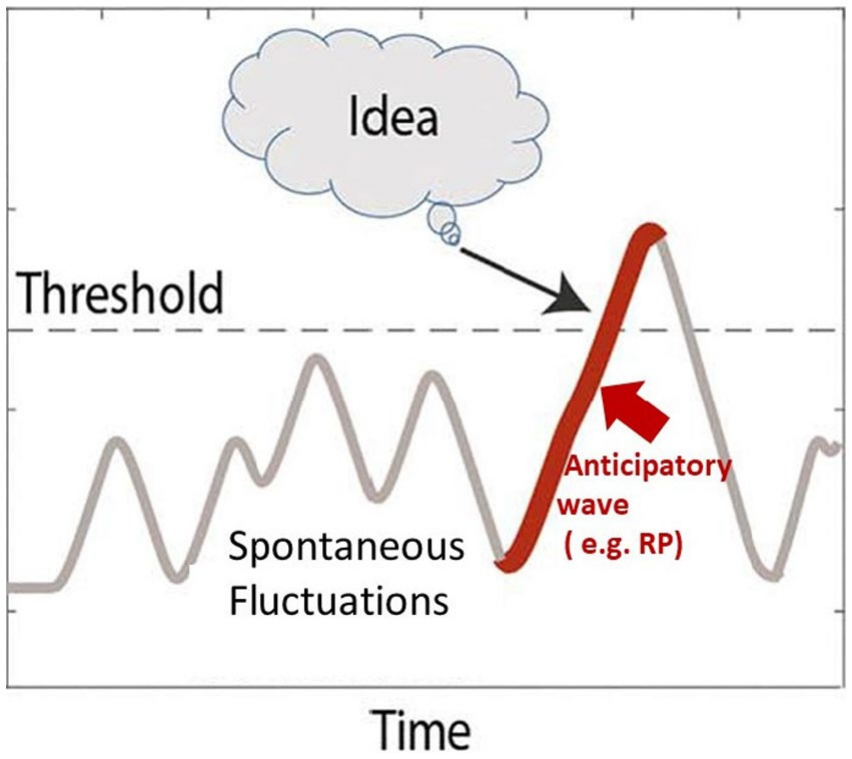
Experimental evidence linking spontaneous fluctuations to free and creative behaviors. The figure illustrates spontaneous neuronal fluctuations during the creative incubation period and the transition to an insight moment. As noted, these slow modulations of activity occur below the dynamic ignition and awareness thresholds (dashed line). The finding of a significant creative solution is implemented as a high-amplitude fluctuation that crosses the awareness threshold. Note that due to the sluggish nature of the spontaneous fluctuations, it is predicted that the anticipatory buildup prior to the moment of creative insight (red line) will be slow (1–3 s), see Broday-Dvir and Malach (2021).

How could spontaneous fluctuations lead to a creative insight? Put simply, the idea is that spontaneous fluctuations constitute an unconscious, semi-random, neuronal search in the space of all possible solutions. When this search happens to land on a significant solution, the neuronal activity crosses the awareness threshold, leading to creative insight. Figure 1 illustrates how it is hypothesized that the process is dynamically manifested in the cortical circuitry (Broday-Dvir and Malach, 2021).

As can be seen, the spontaneous fluctuations introduce slow, semi-random perturbations of the network state, which explore the space of possible solutions. The moment the creative insight reaches awareness is reflected in a transition from the slow undulations of activity that occur below the awareness threshold toward rapid, non-linear, “ignition” dynamics (Fisch et al., 2009) due to the positive feedback inherent in the recurrent nature of cortical circuitry [see also Moutard et al. (2015)].

The transition between the subconscious exploration mode mediated by the spontaneous fluctuations and the rapid ignition is reflected in an upward swing of one spontaneous fluctuation that is simply large enough to cross the network criticality threshold. The process has been successfully simulated in a simple recurrent neuronal network model (see Yellin et al. (2023)). It should be pointed out that this hypothetical dynamic scenario is not a rare or idiosyncratic hypothesis but a common and well-documented process associated with decision-making, pioneered, for example, in the work of Shadlen and Newsome (2001) and later expanded to the domain of voluntary behaviors in the elegant research of Schurger et al. (2012).

So far, it has been demonstrated that spontaneous fluctuations manifest similar properties to those we experience in the creative process, and a plausible mechanism by which they could play a critical role in such a process has been offered. However, one could justifiably ask whether there is experimental evidence that supports this conjecture. Examining Figure 1 highlights two empirically verifiable predictions that can be derived from this model.

**Figure 2:**
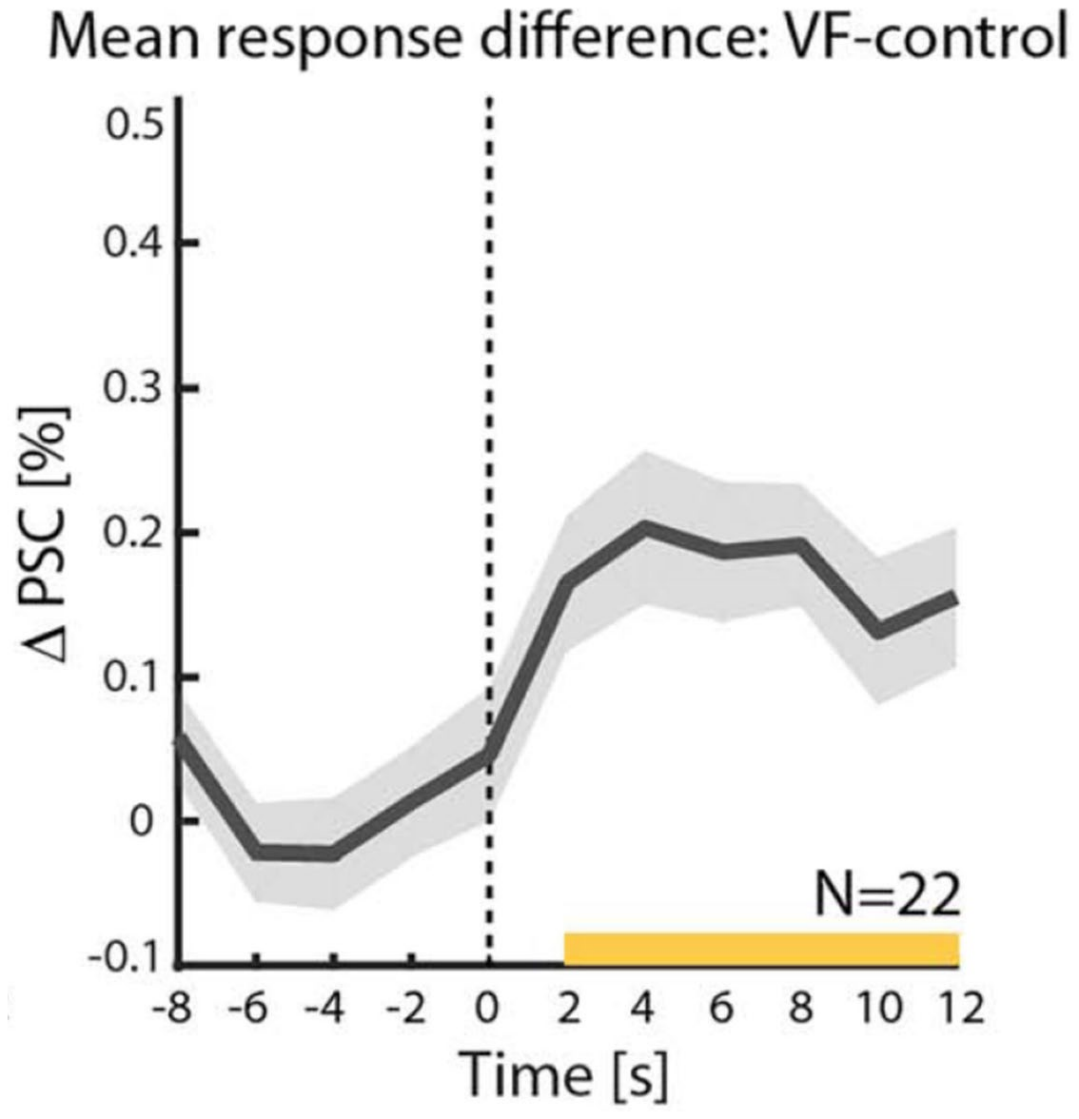
Slow buildup prior to a creative decision (verbal fluency task). Shown is the incremental BOLD fMRI activation during a creative insight task over control. The signal is time-locked to the moment of creative insight (dashed line). The ordinate depicts the difference between BOLD activation during a verbal fluency task in which participants were asked to come up with as many names beginning with a certain letter as they could and a word repetition control in which the participants repeated a name at the same temporal sequence as the verbal frequency task. Note the significant anticipatory buildup associated with the reported moment of insight. For more details see Broday-Dvir and Malach (2021).

First, as noted above and illustrated by the red line, due to the sluggish nature of the spontaneous fluctuations, each “aha” moment of creative insight is expected to be preceded by a slow buildup, which is nothing more than the rising phase of a single high amplitude fluctuation that happens to cross the awareness threshold. Fortunately, due to their slow dynamics, extending typically over seconds [see also Moutard et al. (2015)], these anticipatory buildups can be detected using fMRI. Indeed, a recent study demonstrated this slow buildup in the case of two kinds of verbal creative moments (Broday-Dvir and Malach, 2021). This finding is illustrated in Figure 2.

However, it could be argued that a slow buildup prior to decision-making is a common feature that has been shown in many situations, in particular, when the evidence is noisy and needs to be accumulated prior to such decisions [e.g., Shadlen and Newsome (2001)]. In order to demonstrate, experimentally, that creative decisions are driven by ultra-slow spontaneous fluctuations, a link must be demonstrated between these two, seemingly unrelated, phenomena.

**Figure 3:**
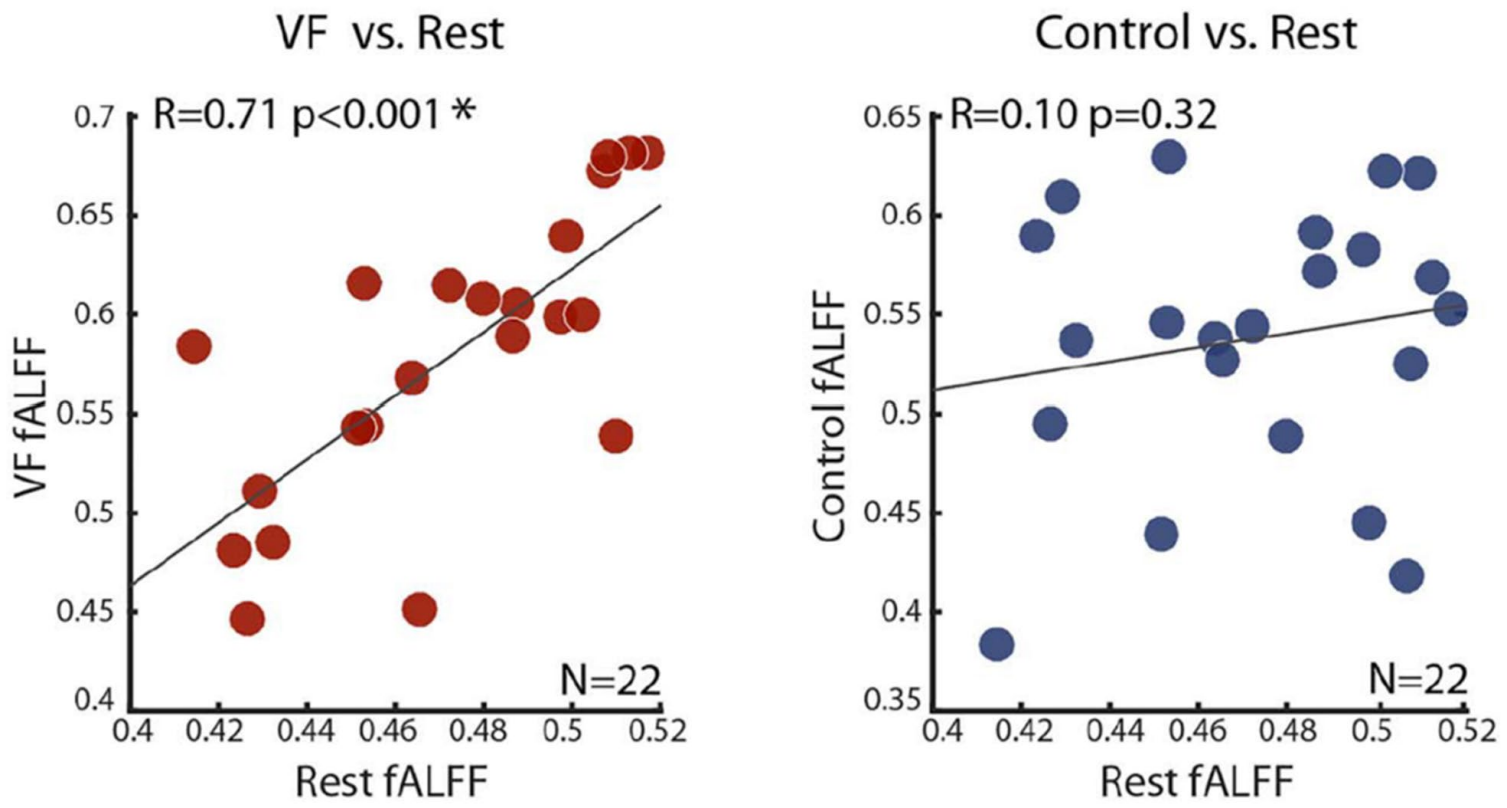
Spontaneous fluctuations are correlated to the signal buildup prior to creative moments. The scatter plots depict the correlation between the dynamic slopes of the spontaneous activity fluctuations measured during rest (x-axis) and the slope of the anticipatory buildup found prior to creative moments (y-axis, left panel, visual fluency task). Compare this correlation to the word repetition control (y-axis, right panel). Note the significant correlation specific to the creative tasks but not for the control tasks [see Broday-Dvir and Malach (2021) for details].

An opportunity to demonstrate such a link is provided by the finding that the dynamics of the spontaneous fluctuations, and in particular, their power spectra, vary across cortical regions and individuals. Thus, some individuals have more sluggish, slowly rising, spontaneous fluctuations, while others have choppier ones. If indeed the anticipatory wave prior to creative decisions is a single, large, spontaneous fluctuation, we would expect the slope of the anticipatory wave to correspond to the slope of the spontaneous fluctuations. Indeed, this was examined and proven to be the case in the Borday-Dvir study (Broday-Dvir and Malach, 2021) and is illustrated in Figure 3. Thus, a direct link has been demonstrated between spontaneous fluctuations and the initiation of creative moments.

### Properties of spontaneous fluctuations account for well-known properties of the creative process

Creativity depends on a rich and complex set of factors. The following section focuses on three well-established aspects of the creative process and shows how known properties of spontaneous fluctuations may provide a neurocognitive explanation of these properties.

### A. Creativity reflects the underlying knowledge, experience, and personality traits of the individual

As has been amply demonstrated throughout history, for example in Mozart vs. Einstein, it is obvious that human individuals greatly differ in the nature and extent of their creative domains. The creative force can be manifested in widely disparate fields, ranging from music to art, science, and technological innovation. As we argue here that spontaneous fluctuations constitute a universal mechanism that underlies creativity, one may ask in what way different individuals differ in their spontaneous fluctuations and what factors shape these differences.

Indeed, a massive body of research has already specifically addressed this question, focusing on what appears to be the most individually unique signature of spontaneous fluctuations, namely, their dynamically changing co-activation patterns. These patterns are commonly studied by searching for correlated activations between pairs of cortical sites typically called “functional connectivity.” A full survey of this literature is beyond the scope of the current review; however, the main factors that appear to play a significant role in shaping the spontaneously emerging activation patterns are prior experiences, task-specific training, and personality traits (Harmelech and Malach, 2013). In the context of the present review, these findings establish a direct link between the acquired knowledge, expertise, and personal history of an individual and their creative abilities.

To illustrate this link through one particularly interesting example, we can take the case of long-term mindfulness meditators. This kind of meditation emphasizes focusing attention on the here and now, rather than the habitual attention to past and future concerns. We have previously argued that such attention to the moment-by-moment experience enhances externally oriented networks, such as visuomotor networks, and inhibits self and memory-related networks such as medial pre-frontal and default mode networks (Goldberg et al., 2006; Golland et al., 2007). In line with our hypothesis linking habitual activation of cortical networks with the spontaneous re-emergence of such networks during rest, we would expect that in long-term mindfulness meditators, and in accordance with their long-term training, their spontaneous fluctuations will show enhanced power in their visuomotor networks and a concomitant reduction in power in memory-related networks such as the default mode network. Indeed, in an extensive research study of spontaneous fluctuations, long-term mindfulness meditators exhibited a significantly higher power in visual networks compared to default mode networks (Berkovich-Ohana et al., 2013). This finding, in turn, leads to the prediction that long-term meditators should show enhanced creativity in moment-by-moment behaviors, which will be an interesting topic to explore in future studies.

More generally, the experience- and training-dependent shaping of a spontaneously emerging network is nicely compatible with the well-established fact that creativity depends on deep expertise and training in the specific field in which the individual’s creative power is expressed and provides a neurophysiological mechanism that implements such link.

### B. Creativity requires willful effort

Although we often feel that our creative insights appear “out of the blue,” they invariably follow a period of intense and willful, often frustrating, search. Furthermore, we have easy and flexible control over the domain in which our creative insight will be expressed. A musician who intends to write a new symphony will not invent a mathematical theorem by mistake. A choreographer will create a new dance piece and not a poem and so forth. This control is so easily executed that we take it for granted. However, without such an initial and well-focused drive to find a solution within a specific field, no creative insight will emerge. Thus, in the context of the spontaneous fluctuations hypothesis, it is of interest to ask whether willful effort can both focus and amplify the power of spontaneous fluctuations and, through this, enhance the probability of a creative act occurring specifically within the targeted domain.

**Figure 4:**
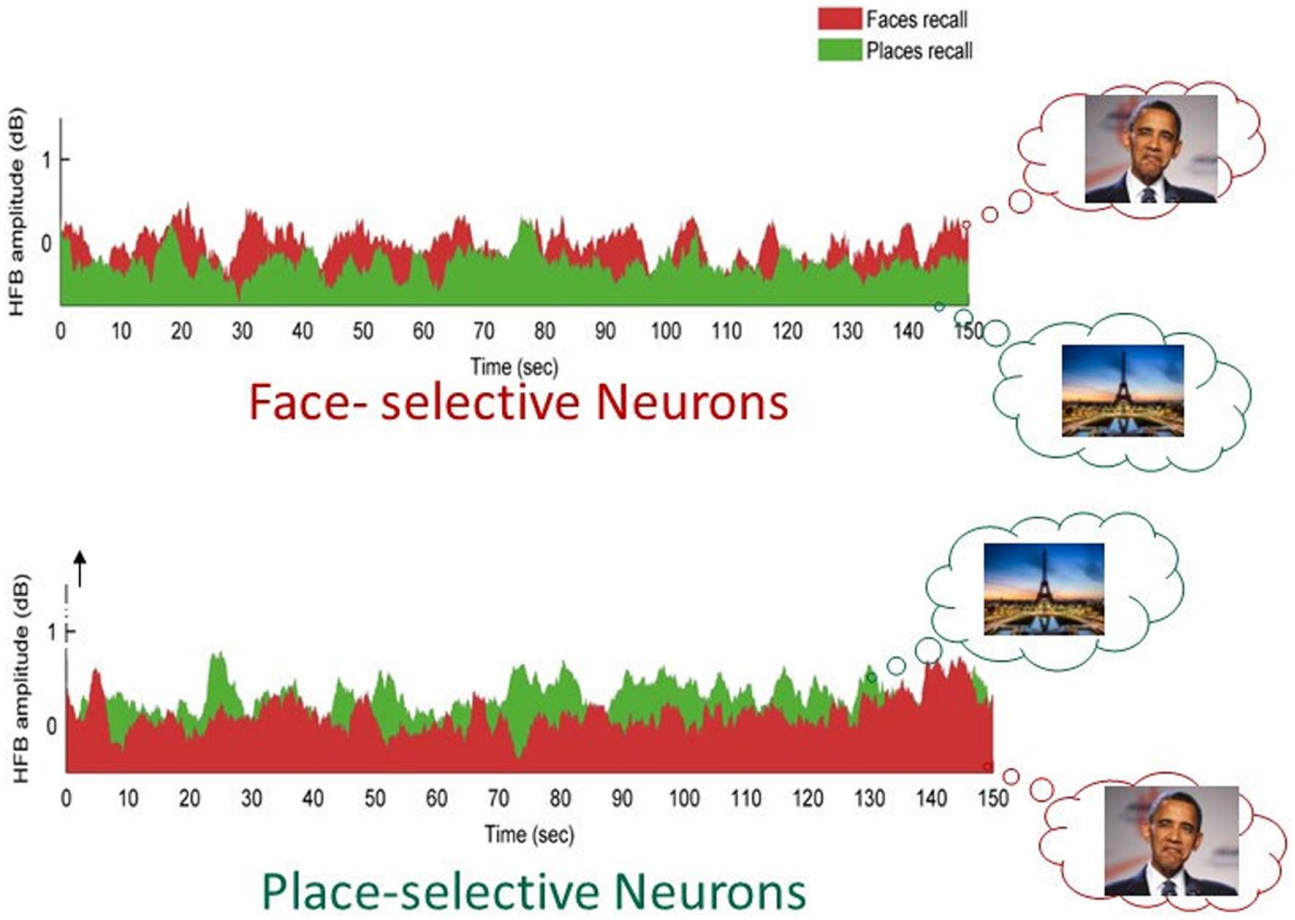
Modulation of spontaneous fluctuations by willful effort. Plots of intracranially recorded signals were obtained from face-selective and place-selective cortical regions, respectively, during a free recall task in which patients attempted to freely recall faces (red) or places (green). Note that attempting to recall faces or places resulted in enhanced spontaneous activity fluctuations in the face-selective or place-selective neurons, respectively. These findings highlight a neuronal mechanism allowing willful control of spontaneous fluctuation levels in the human cortex. Images reproduced with permission from the author, available at Norman et al. (2017).

This issue was examined experimentally by Norman et al. (2017), who studied targeted, category-specific, free visual recall. In the experiment, patients implanted intracranially with intracranial electroencephalography (IEEG) electrodes for clinical purposes, were asked to freely recall, in separate sessions, either famous faces or famous places. Figure 4 depicts the results of this experiment. As can be seen, IEEG contacts located in face-selective cortical regions showed enhanced spontaneous fluctuations that were both shifted toward the decision threshold and showed increased amplitude of the spontaneous fluctuations. Critically, this amplification was domain-specific: In the face-selective regions, the amplification occurred when patients attempted to recall faces but not when they tried to recall places and vice versa for the place-selective regions. Thus, the amplitude of spontaneous fluctuations can be flexibly and rapidly modulated in a category-specific manner. This offers a powerful mechanism for individuals to direct their creative efforts toward specific and well-defined fields.

### C. Creativity employs stochasticity

When considering how spontaneous fluctuations contribute to the creative process, a useful metaphor illustrating such a role could be derived from the field of optimization methods where the aim is to find an optimal solution to a complex problem. The process is typically visualized as a ball rolling on a surface where dents represent possible solutions, with relatively good solutions portrayed as deeper dips on this surface. The problem is that left to the pull of gravity, the ball will settle in an occasional shallow dent and arrive at a sub-optimal solution. However, if the ball is bombarded by spontaneous noise, it will dislodge from the shallow dent and keep rolling until it arrives at the deepest well from which the noise cannot extricate it [see, e.g., Aarts et al. (2006)].

In this metaphor, the spontaneous fluctuations play the role of the noise; in other words, by randomly activating the relevant neuronal networks, they enlarge the exploration of possible network states, allowing the discovery of an optimal creative insight. Hitting upon such an insight is instantiated, neuronally, as a large amplitude increase in the network activity—moving the network from its below-threshold, subconscious, search state to crossing the critical threshold reflected in a neuronal “ignition” [see Moutard et al. (2015)] experienced as an “aha” moment in which the individual becomes aware of the creative insight.

The first two aspects of creativity discussed above emphasize the deterministic control individuals have over their creative process—through long-term training and experience on the one hand and short-term effort and will on the other. Here, it is proposed that creativity also necessitates an element of randomness, of stochasticity, which contributes a critical element of the unexpected, “out of the box,” to the creative process. Can such an element of stochasticity be found in spontaneous fluctuations? Although the source of spontaneous fluctuations is still unknown, it is likely that their origin stems from neuronal noise, which is abundant at the cellular level in each and every neuron. Indeed, spontaneous fluctuations can be observed in any neuronal system, including small groups of neurons grown in a dish (Penn et al., 2016).

In a recent modeling study (Yellin et al., 2023), (Modulation of proximity to criticality enhances slow activity fluctuations during free recall, Yellin, N. Siegel, R. Malach, O. Shriki bioRxiv 2023.02.24) showed that a simple recurrent network receiving random synaptic inputs can readily exhibit ultra-slow fluctuations whose dynamics mimic the spontaneous fluctuations recorded in the human cortex. The basis for this transformation of fast stochastic noise into ultra-slow fluctuations is a well-known property of recurrent networks, termed slowing down criticality, in which these networks exhibit slow dynamics as they approach the phase transition threshold. Furthermore, in the simulation, the randomly activated network exhibited the experimentally observed property of targeted amplification shown by Norman et al. (2017) when the network was uniformly activated.

## In summary

I propose here a universal mechanism that underlies the uniquely human property of creative insight: ultra-slow spontaneous fluctuations of neuronal activity. This mechanism fulfills the central properties of the creative process, its diversity, its dependence on willful effort and prior expertise, and its self-generated nature. Experimental evidence links spontaneous fluctuations of the human brain to the anticipatory buildup observed across all free and creative behaviors.

Examining the properties of spontaneous fluctuations reveals that they form an intriguing case of biased stochasticity, in which both randomness and deterministic information play a role. One cannot but notice the parallels between the creative process and biological evolution in which randomly generated mutations in the genetic material combine with its inherited deterministic order to form novel species and biological structures. In both human creativity and biological evolution, the integration of randomness and order leads to stunningly successful outcomes.

## Author contributions

RM: Writing – original draft, Writing – review & editing.

## References

[ref1] Aarts E. van der Horn P. Korst J. Michiels W. Sontrop H. (2006). Simulated annealing. Oper. Res. Comput. Sci. 36, 37–52.

[ref2] Arieli A. Sterkin A. Grinvald A. Aertsen A. (1996). Dynamics of ongoing activity: explanation of the large variability in evoked cortical responses. Science 273, 1868–1871. doi: 10.1126/science.273.5283.1868, PMID: 8791593

[ref3] Berkovich-Ohana A. Arieli A. Malach R. (2013). Decreased "default-mode" activity in trained meditators. J. Mol. Neurosci. 51, S20–S21.

[ref4] Broday-Dvir R. Malach R. (2021). Resting-state fluctuations underlie free and creative verbal behaviors in the human brain. Cereb. Cortex 31, 213–232. doi: 10.1093/cercor/bhaa221, PMID: 32935840

[ref5] Fisch L. Privman E. Ramot M. Harel M. Nir Y. Kipervasser S.. (2009). Neural "ignition": enhanced activation linked to perceptual awareness in human ventral stream visual cortex. Neuron 64, 562–574. doi: 10.1016/j.neuron.2009.11.001, PMID: 19945397 PMC2854160

[ref6] Fox M. D. Raichle M. E. (2007). Spontaneous fluctuations in brain activity observed with functional magnetic resonance imaging. Nat. Rev. Neurosci. 8, 700–711. doi: 10.1038/nrn2201 17704812

[ref7] Goldberg I. I. Harel M. Malach R. (2006). When the brain loses its self: prefrontal inactivation during sensorimotor processing. Neuron 50, 329–339. doi: 10.1016/j.neuron.2006.03.015, PMID: 16630842

[ref8] Golland Y. Bentin S. Gelbard H. Benjamini Y. Heller R. Nir Y.. (2007). Extrinsic and intrinsic systems in the posterior cortex of the human brain revealed during natural sensory stimulation. Cereb. Cortex 17, 766–777. doi: 10.1093/cercor/bhk030, PMID: 16699080

[ref9] Harmelech T. Malach R. (2013). Neurocognitive biases and the patterns of spontaneous correlations in the human cortex. Trends Cogn. Sci. 17, 606–615. doi: 10.1016/j.tics.2013.09.014 24182697

[ref10] Moutard C. Dehaene S. Malach R. (2015). Spontaneous fluctuations and non-linear ignitions: two dynamic faces of cortical recurrent loops. Neuron 88, 194–206. doi: 10.1016/j.neuron.2015.09.018, PMID: 26447581

[ref11] Nir Y. Hasson U. Levy I. Yeshurun Y. Malach R. (2006). Widespread functional connectivity and fMR1 fluctuations in human visual cortex in the absence of visual stimulation. NeuroImage 30, 1313–1324. doi: 10.1016/j.neuroimage.2005.11.018 16413791

[ref12] Nir Y. Mukamel R. Dinstein I. Privman E. Harel M. Fisch L.. (2008). Interhemispheric correlations of slow spontaneous neuronal fluctuations revealed in human sensory cortex. Nat. Neurosci. 11, 1100–1108. doi: 10.1038/nn.2177, PMID: 19160509 PMC2642673

[ref13] Norman Y. Yeagle E. M. Harel M. Mehta A. D. Malach R. (2017). Neuronal baseline shifts underlying boundary setting during free recall. Nat. Commun. 8:1301. doi: 10.1038/s41467-017-01184-1, PMID: 29101322 PMC5670232

[ref14] Penn Y. Segal M. Moses E. (2016). Network synchronization in hippocampal neurons. Proc. Natl. Acad. Sci. USA 113, 3341–3346. doi: 10.1073/pnas.1515105113, PMID: 26961000 PMC4812773

[ref15] Ramot M. Grossman S. Friedman D. Malach R. (2016). Covert neurofeedback without awareness shapes cortical network spontaneous connectivity. Proc. Natl. Acad. Sci. USA 113, E2413–E2420. doi: 10.1073/pnas.1516857113, PMID: 27071084 PMC4855583

[ref16] Ramot M. Wilf M. Goldberg H. Weiss T. Deouell L. Y. Malach R. (2011). Coupling between spontaneous (resting state) fMRI fluctuations and human oculo-motor activity. NeuroImage 58, 213–225. doi: 10.1016/j.neuroimage.2011.06.015, PMID: 21703354

[ref17] Schurger A. Sitt J. D. Dehaene S. (2012). An accumulator model for spontaneous neural activity prior to self-initiated movement. Proc. Natl. Acad. Sci. USA 109, E2904–E2913. doi: 10.1073/pnas.1210467109, PMID: 22869750 PMC3479453

[ref18] Shadlen M. N. Newsome W. T. (2001). Neural basis of a perceptual decision in the parietal cortex (area LIP) of the rhesus monkey. J. Neurophysiol. 86, 1916–1936. doi: 10.1152/jn.2001.86.4.1916, PMID: 11600651

[ref19] Smith S. M. Fox P. T. Miller K. L. Glahn D. C. Fox P. M. Mackay C. E.. (2009). Correspondence of the brain's functional architecture during activation and rest. Proc. Natl. Acad. Sci. USA 106, 13040–13045. doi: 10.1073/pnas.0905267106, PMID: 19620724 PMC2722273

[ref001] Yellin D. Malach R. Shriki O. (2023). Modulation of proximity to criticality enhances slow activity fluctuations during free recall bioRxiv. 2023–2., PMID: 22869750

